# Effects of Supraorbital Foramen Variations on the Treatment Efficacy of Radiofrequency Therapy for V1 Trigeminal Neuralgia: A Retrospective Study

**DOI:** 10.1155/2020/8142489

**Published:** 2020-02-26

**Authors:** Keyue Xie, Songlei Liu, Bing Huang, Ming Yao

**Affiliations:** ^1^Department of Anesthesiology and Pain Medicine, First Affiliated Hospital of Jiaxing University, Jiaxing, China; ^2^Bengbu Medical College Graduate Department, Bengbu Medical College, Bengbu, China

## Abstract

**Background:**

Primary V1 trigeminal neuralgia is a common refractory neuralgia in clinical practice, lacking effective treatments. Radiofrequency therapy has certain treatment efficacy, but its long-term efficacy remained poor and the disease might relapse.

**Objective:**

To compare the effects of different types of supraorbital foramen variations on the treatment efficacy of radiofrequency therapy for V1 trigeminal neuralgia.

**Methods:**

Data of 54 patients with V1 trigeminal neuralgia who underwent treatment in the First Hospital of Jiaxing, Zhejiang, were retrospectively analyzed. All these patients received CT-guided radiofrequency thermocoagulation of supraorbital nerve. According to the CT images, the supraorbital foramen of the patients was categorized as holes (hole group) or notches (notch group). The patient characteristics, including Numerical Rating Scale (NRS) score and effective treatment rates before and 1 d, 0.5 y, 1 y, and 2 y after operation, and numbness degree at day 1 and 2 y after the operation were compared. The short- and long-term complications during postoperative follow-up period were also recorded.

**Results:**

Among the 54 patients, 25 patients were grouped into the hole group and 29 into the notch group. The NRS scores before and at 1 d, 0.5 y, 1 y, and 2 y after operation showed no significant differences between the two groups. However, the NRS scores at the remaining time points after operation were significantly decreased when compared with scores before operation (*P* < 0.05). The numbness and numbness degree after operation showed no significant differences between the two groups. The numbness degree at 2 y after operation was significantly lower than 1 d after operation (*P* < 0.05). The numbness and numbness degree after operation showed no significant differences between the two groups. The numbness degree at 2 y after operation was significantly lower than 1 d after operation (*P* < 0.05). The numbness and numbness degree after operation showed no significant differences between the two groups. The numbness degree at 2 y after operation was significantly lower than 1 d after operation (

**Conclusion:**

The short- and long-term effective rates of radiofrequency therapy during V1 trigeminal neuralgia treatment are relatively high in patients with different types of supraorbital foramen variations. However, the effective rate is even higher in patients with hole-type supraorbital foramen. No other severe complications, except numbness, were found, and the acceptability rate remained high in patients.

## 1. Introduction

The primary trigeminal neuralgia is mainly manifested as paroxysmal shock-like intense pain, restricting the areas innervated by the trigeminal nerve [1]. The pain attack could occur at any time, and the resulting intense pain severely affects the quality of life of the patients. Long-term effects of trigeminal neuralgia include anxiety, depression, and even suicide [2]. The pathological processes of primary trigeminal neuralgia are very complex, and the pathogeneses are still unclear. Till date, there are no effective treatments available for the treatment of trigeminal neuralgia.

Radiofrequency treatment for trigeminal neuralgia has shown definitive treatment efficacies [3]. However, with conventional radiofrequency therapy of Gasserian ganglion through foramen ovale, the sense and movement in the areas innervated by the ophthalmic nerve, which is the first branch of trigeminal nerve, may not be detected despite perfect puncturing, leading to severe complications, such as blindness induced by corneal ulcer [4]. To prevent severe complications, radiofrequency therapy at low temperature (42–60°C) is generally applied in clinical practice. However, the effect of treatment rate is not high, and the effective duration is not long, thus requiring repeated therapies. Therefore, our study group has conducted continuous standard radiofrequency thermocoagulation (95°C) through supraorbital foramen puncture under the guidance of CT for treating primary V1 trigeminal neuralgia to ensure treatment efficacy while preventing severe complications [5].

Although the peripheral supraorbital nerve resection is done at high-temperature radiofrequency, relapse still occurred in some patients. To increase the duration of trans-supraorbital foramen radiofrequency therapy on primary trigeminal neuralgia and reduce the relapse rate effectively, this study retrospectively analyzed the data of 54 patients with primary V1 trigeminal neuralgia who underwent treatment between November 2011 and June 2017 to provide evidence. After the analysis of long-term follow-up data, as well as the imaging findings during the operation, the findings revealed that different shapes of supraorbital foramina could affect the destruction of the nerves by radiofrequency therapy, as well as the velocity of nerve regeneration after operation. This study aimed to compare the influences of different supraorbital foramen variations on the treatment efficacies of radiofrequency therapy in patients with primary V1 trigeminal neuralgia.

## 2. Subjects and Methods

### 2.1. Subjects

Fifty-four patients with primary V1 trigeminal neuralgia who received CT-guided radiofrequency thermocoagulation through the supraorbital foramen in the First Hospital of Jiaxing, Zhejiang, between November 2011 and June 2017 were included. All patients included in this study met the diagnostic criteria of primary trigeminal neuralgia as described in the International Classification of Headache Disorders (3rd edition) [6]. The inclusion criteria were as follows: (1) patients aged >18 years; (2) patients with only primary V1 trigeminal neuralgia, or combined with V2/V3 trigeminal neuralgia; (3) patients with preoperative Numerical Rating Scale (NRS) score of >5; (4) patients with disease course of >1 month; and (5) patients whose pain was not relieved after treatment with carbamazepine, gabapentin, or pregabalin alone or in combination, or the patient could not tolerate the adverse responses. The exclusion criteria were as follows: patients (1) who undergoes routine CT scanning of the paranasal sinus and MRI examinations of trigeminal nerve before the operation suggesting secondary trigeminal neuralgia; (2) with trigeminal neuralgia secondary to herpes zoster; (3) with local infection at the site of puncture; (4) with psychiatric disorders or intellectual disability; (5) with severe liver, kidney, heart, or lung diseases; and (6) with coagulation disorders before operation, or allergic to local anesthetics. All the surgical protocols were approved by the Ethics Committee of the First Hospital of Jiaxing. The patients or the families signed the informed consent forms.

### 2.2. Methods

**Figure 1:**
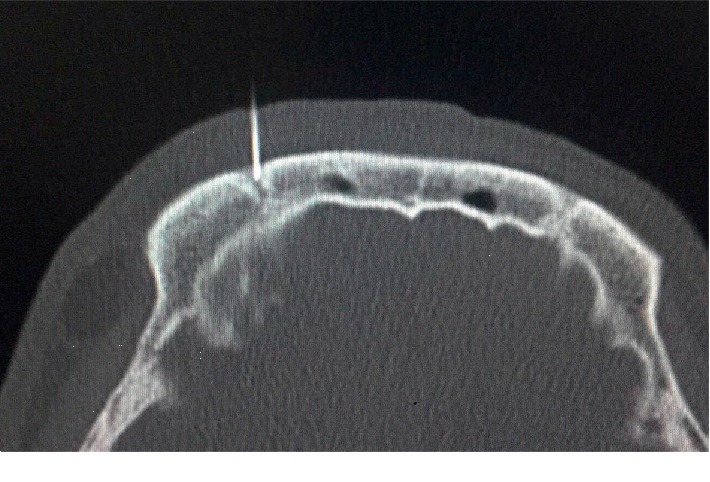
Insertion of puncture needle into the supraorbital foramen under the guidance of CT.

The patients were placed in the supine position on the table for undergoing CT treatment, with the head fixed by the tape. The site and route of puncture were designed by CT software to measure the depth of punctuation. The puncture site was then marked on the body surface. After local anesthesia was induced by 1% lidocaine, a 22 G radiofrequency puncture needle with a bare segment length of 2 mm was used for puncturing at the predefined site and predefined depth. The needle was then adjusted according to the CT scanning results until the needle point reaches the supraorbital foramen (Figure 1). After the puncture was completed, the radiofrequency puncture needle was fixed, the stylet was withdrawn, and the radiofrequency electrode was inserted for impedance tests, sensory and movement at high and low frequencies, and electrophysiology. If 0.5 mA or lower currency could induce pain in the area of primary lesions in sensory tests (high frequency, 50 Hz, 0.1 V) and myoclonus in the ipsilateral supraorbital area in the movement test (low frequency, 2 Hz, 1.0 V), the target radiofrequency was then considered accurate. After that, 1.5–2.0 mg/Kg propofol was intravenously injected for anesthesia induction, and then, continuous standard radiofrequency thermocoagulation (90°C, 120 s) was performed. After the patient was awaken, the sensory changes in the areas innervated by the ipsilateral ophthalmic nerve of trigeminal nerve were tested until the pain disappeared. Otherwise, the position of the needle point was adjusted, and then, the test and radiofrequency thermocoagulation were conducted again. After the radiofrequency therapy was completed, the patient was placed in the supine position and observed for 30 min and then returned to the ward if no discomfort was reported. All the operations were conducted by the same senior chief surgeon.

The characteristics of the patients, including age, gender, disease course, times of CT scanning, NRS (range from 1 to 10, reflecting the severity of pain) score, and effective rate (NRS ≤1 was considered effective) before and on day 1, at 0.5 y, 1 y, and 2 y after operation, as well as the numbness degree (barrow degree: grade I indicated no numbness in the face; grade II indicated mild facial numbness, which was not annoying; grade III indicated facial numbness, which was annoying occasionally; and grade IV indicated facial numbness, which was very annoying) on day 1 and at 2 y after the operation, and the shapes of the supraorbital foramen were obtained through the hospital information systems (HIS) medical record system or telephone call. The short- and long-term complications after the operation were also recorded by telephone follow-up.

### 2.3. Statistical Analysis

SPSS 24.0 software was used for statistical analysis. The quantitative data that are normally distributed were described as means ± standard divisions ($\bar{x}\pm s$). The independent *t*-test was used for comparisons between groups, while the paired *t*-test was used for comparisons between different batches. Qualitative data were described with frequencies and percentages (%) and compared with chi-square (*χ*^2^) test. *P* < 0.05 was considered to be statistically significant.

## 3. Results

### 3.1. General Characteristics

**Figure 2:**
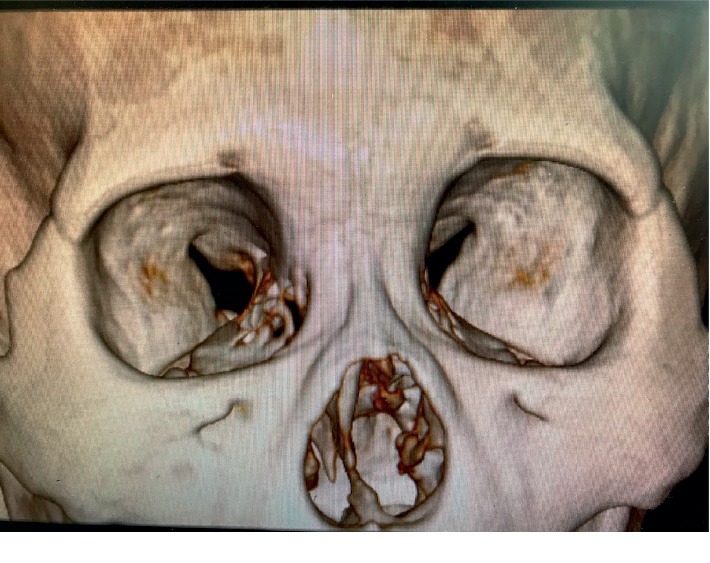
Hole-type supraorbital foramen.

**Figure 3:**
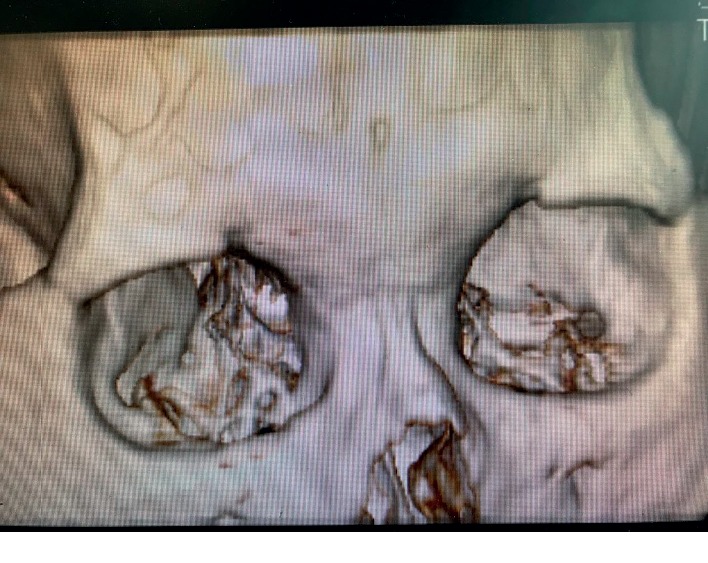
Notch-type supraorbital foramen.

Among the 54 patients included in this study, the shape of the ipsilateral supraorbital foramen was hole type in 25 patients (Figure 2) and was notch type in 29 patients (Figure 3). Age, gender, disease course, times of CT scanning, and follow-up time showed no significant differences between the hole group and notch group (Table 1).

### 3.2. NRS Score at Different Time Points

The NRS scores before and at day 1, 0.5 y, 1 y, and 2 y after the operation showed no significant differences between the two groups (*P* > 0.05). However, comparison of NRS scores before operation and at the remaining time points showed a significant decrease in both groups (*P* < 0.05, Table 2).

### 3.3. Numbness Degree

The numbness degrees at day 1 and 2 y after the operation showed no significant differences between the two groups (*P* > 0.05). However, the numbness degree at 2 y after the operation was significantly lower than at day 1 after the operation in both groups (*P* < 0.05, Table 3).

### 3.4. Effective Rate at Different Time Points

When compared with the hole group, the effective rates at day 1, 0.5 y, and 1 y after the operation showed no significant differences (*P* > 0.05). However, the effective rate was significantly lower in the notch group than the hole group at 2 y after operation (*P* < 0.05, Table 4).

### 3.5. Complications

Different degrees of swallowing at the puncture site were found in both groups after the operation, which disappeared spontaneously at 1–3 d without specific treatments. No other short- or long-term complications were found.

## 4. Discussion

In 54 patients with primary V1 trigeminal neuralgia, the supraorbital foramen showed great variations. The supraorbital foramina were mainly categorized into two types, namely, the hole type and the notch type. When compared with patients with the hole-type supraorbital foramen, the numbness degree was generally comparable in the notch group shortly after operation after the same treatments were conducted; however, the recovery of numbness was faster during long-term treatment, and the relapse rate was higher in the notch group. The differences in the effects of this specific anatomic structure on treatment efficacy could be due to several other reasons. For instance, the supraorbital nerve travels through the supraorbital foramen, while the route of travelling was generally unchanged. For patients with hole-type supraorbital foramen, the supraorbital nerve travels through the supraorbital foramen, and thus puncturing at this site for radiofrequency therapy could completely destruct the nerve. While for the patients with notch type supraorbital foramen, the supraorbital nerve may not travel through the supraorbital foramen. But travelling of supraorbital nerve might show some inner or outer deviation. Therefore, such anatomic variations could lead to deviations between the target puncturing site for radiofrequency therapy and actual site of the nerve, consequently leading to a relatively higher relapse rate, and in turn, affecting clinical efficacy [7]. The hole-type supraorbital foramen is the only target for radiofrequency therapy. During the treatment, the physicians insert the needle point into the supraorbital foramen, and the position of the needle is relatively fixed after the puncture is completed. Therefore, the possibility of shifting remained relatively low during puncturing and radiofrequency therapy. However, for patients with notch-type supraorbital foramen, there are no limitations for the targets of radiofrequency therapy. Due to subjective factors from the physician, the needle point may not be fixed on the supraorbital notch after the puncture is completed. In addition, the unconscious movement of the patients, as well as the movement induced by pain stimuli, could increase the risk of shifting the needle point, thus affecting the treatment efficacy. The relapse of trigeminal neuralgia after radiofrequency thermocoagulation is mainly caused due to incomplete destruction of the nerve by radiofrequency therapy, or nerve regeneration. After radiofrequency therapy, the neuropathological changes of the nerve mainly included Wallerian degeneration and axonal regeneration. The nerve fiber disintegrates rapidly during the early phase after therapy. However, the nerve fiber could regenerate, although the neurons could not or only regenerate extremely slowly [8].

Radiofrequency therapy has been widely applied in clinical practice for the treatment of trigeminal neuralgia [9]. Although treatment at different targets as well as using different parameters has efficacy in short-term, the long-term relapse rate and numbness grade varied greatly. In a study conducted by Kim et al., histological examinations showed that low-temperature (42°C) radiofrequency therapy for dorsal root ganglion and sciatic nerve caused temporary endothelial edema and collagen deposition of the nerve, while no structural changes were found [10]. According to a study by Erdine et al., electron microscopy demonstrated the damages of axonal ultrastructure, abnormalities of membrane and mitochondria, and collapse of microtubules and microfilaments after undergoing low-temperature radiofrequency therapy [11]. Tun et al. showed that low-temperature radiofrequency therapy could damage the myelin sheath of the myelin coating axons [12]. However, all these changes are reversible. In a prospective, randomized, double-blind study, the short-term remission rate was only 10% in patients with primary trigeminal neuralgia who received low-temperature (42°C) radiofrequency therapy for Gasserian ganglion. In addition, the relapse was found in all patients 3 months later, and the treatment efficacy remained relatively poor [13]. Yao et al. [14] compared the treatment efficacy of radiofrequency therapy at different temperatures (62°C, 65°C, and 68°C) for trigeminal neuralgia, and the results revealed that the relapse rates were significantly higher in 62°C and 65°C groups than in the 68°C group. These findings suggested that with high temperature of radiofrequency therapy, the relapse rate remained low, and the numbness degree remained high [15–17]. In this study, high temperature (95°C) was adopted for radiofrequency therapy to reduce thermal attenuation caused by the distance from the radiofrequency target and target nerve, which in turn leads to incomplete nerve destruction and high relapse rate. In addition, the room in the notch-type supraorbital foramen is larger than hole type; thus, the connective tissues in the room could disperse the temperature of the radiofrequency therapy, consequently leading to incomplete destruction of the nerve [18]. High temperature could induce cellular apoptosis and necrosis. The damage caused at high temperatures of 95°C could evidently damage the neurons and nerve fibers [19, 20]. Active damaging of the nerves for the treatment of some diseases has been highly accepted by the patients. The definition of “effective” for the treatment of trigeminal neuralgia varied in different studies [21–23]. For instance, some studies have defined “effective” as pain remission by >50% after treatment [24, 25]. However, the primary trigeminal neuralgia manifests as typical neuropathological pain, mainly as paroxysmal shock-like intense pain which is characterized as “all-or-none.” Therefore, the term “effective” should not be defined by partial remission. We speculated that the treatment efficacy of trigeminal neuralgia should be assessed by complete disappearance of the pain, and if pain still exists after operation, the treatment should be considered as failure. Therefore, NRS score ≤1 after operation was considered effective in this study for treating trigeminal neuralgia.

Compared with Gasserian ganglion, the target for radiofrequency therapy for primary V1 trigeminal neuralgia through supraorbital foramen is closer to the surrounding tissues. Thus, the effect on the branches of some small nerves could be insufficient, and so a part of neuralgia might be missed. The Gasserian ganglion could not be completely destroyed by high-temperature radiofrequency, which might be due to severe complications induced by adjacent nerve damage. More studies are needed to identify new targets and parameters of radiofrequency therapy that are close to the central but cannot easily damage the normal nerves.

## 5. Conclusion

In summary, the short- and long-term effective rates of radiofrequency therapy for treating V1 trigeminal neuralgia are relatively high in patients with different types of supraorbital foramen variations; however, the effective rate is even higher in patients with hole-type supraorbital foramen. No other severe complications, except numbness, were found, and the acceptability remained high in these patients. The sample size with primary V1 trigeminal neuralgia included in this study was relatively small, as this is a single-center study. Our future study should increase the sample size to explore the efficacy of different operation methods in patients with different types of anatomic variations of supraorbital foramen, thus helping to improve the treatment efficacy. We speculated that the use of transverse puncture to increase the destruction area by radiofrequency thermocoagulation might result in better treatment efficacy in patients with notch-type supraorbital foramen than conventional vertical puncture.

## Figures and Tables

**Table 1:** Comparison of general characteristics of patients in the two groups.

	Hole group (*n* = 25)	Notch group (*n* = 29)	*t*/*χ*^2^	*P*
Age (years, $\bar{x}\pm s$)	70.16 ± 14.54	65.31 ± 10.80	1.404	0.166
Gender (M/F)	8/17	14/15	1.473	0.225
Disease course (months, $\bar{x}\pm s$)	42.18 ± 53.44	63.11 ± 68.40	−1.238	0.221
Times of scanning ($\bar{x}\pm s$)	4.56 ± 2.35	3.97 ± 2.16	0.961	0.341
Times of operation ($\bar{x}\pm s$)	1.04 ± 0.20	1.00 ± 0.00	1.079	0.286

**Table 2:** Comparison of NRS scores between the two groups before and at different time points after operation.

Time	Hole group	Notch group	*T*	*P*
Before operation	6.24 ± 0.52	6.34 ± 0.72	−0.603	0.549
1 d	0.60 ± 0.50	0.52 ± 0.50	0.600	0.550
0.5 y	0.32 ± 0.75	0.59 ± 0.95	−1.134	0.262
1 y	0.60 ± 1.19	1.07 ± 1.44	−1.293	0.202
2 y	0.96 ± 1.37	1.69 ± 1.69	−1.723	0.091

**Table 3:** Numbness degree on day 1 and at 2 y after operation between the two groups (*n*(%)).

Time	Hole group (*n* = 25)	Notch group (*n* = 29)	*χ* ^2^	*P* (between the two groups)
1 d after operation			2.782	0.356
I	0 (0%)	0 (0%)		
II	15 (60%)	5 (17.2%)		
III	10 (40%)	23 (79.3%)		
IV	0 (0%)	1 (3.4%)		
2 y after operation			2.581	0.461
I	11 (44%)	17 (58.6%)		
II	11 (44%)	10 (34.4%)		
III	3 (12%)	2 (6.9%)		
IV	0 (0%)	0 (0%)		
*χ*2	16.697	39.237		
*P*	<0.001	<0.001		

**Table 4:** Comparison of the effective rate between the two groups at different time points after operation (*n*(%)).

	Hole (*n* = 25)	Notch (*n* = 29)	*χ* ^2^	*P*
1 d	25 (100.0)	29 (100.0)	—	—
0.5 y	23 (92.0)	24 (82.6)	1.016	0.313
1 y	20 (80.0)	19 (65.5)	1.404	0.236
2 y	19 (76.0)	14 (48.2)	4.342	0.037

## Abbreviations

CT: Computed tomography
NRS: Numerical Rating Scale.
